# Exercise Load Variation at Different Subjective Intensities of High-Intensity Interval Training Using Body Weight

**DOI:** 10.7759/cureus.78595

**Published:** 2025-02-05

**Authors:** Shojiro Egoshi, Takumi Kandori, Maya Ueno, Manaka Shinya, Aya Wakiji, Rento Ito

**Affiliations:** 1 Faculty of Health Sciences, Hiroshima Cosmopolitan University, Hiroshima, JPN; 2 Department of Rehabilitation, Island Hospital Otani, Etajima, JPN; 3 Department of Rehabilitation, Itsukaichi Memorial Hospital, Hiroshima, JPN; 4 Department of Rehabilitation, Takanobashi Central Hospital, Hiroshima, JPN; 5 Department of Nursing, Merry Hospital, Hiroshima, JPN

**Keywords:** exercise modality, exercise therapy, heart rate, home exercise, oxygen uptake

## Abstract

Introduction: High-intensity interval training (HIIT) using body weight is useful for improving physical function. However, it is unclear how much exercise load can be achieved depending on subjective intensity. This study aims to examine the extent to which body weight exercise at different subjective intensities produces an exercise load according to exercise type in young, healthy adults.

Methods: The participants were 28 young healthy male adults, who performed a progressive exercise load test, and peak oxygen uptake $\grave{V}O_2$ was measured. $\grave{V}O_2$, heart rate (HR), and exercise frequency at each subjective intensity during HIIT were also measured, compared, and verified.

Results: $\grave{V}O_2$ during high-intensity exercises increased significantly with increasing subjective intensity in squats, side lunges, front lunges, and fast walking (p<0.01). However, in squats and fast walking, no significant differences were found between somewhat hard and hard. More than 60% of peak $\grave{V}O_2$ was achieved in squats, side lunges, and front lunges at a subjective intensity of somewhat hard, but only 60% of peak $\grave{V}O_2$ was achieved in fast walking at a hard subjective intensity.

Conclusions: Exercise modalities under body weight, squats, front lunges, and side lunges can produce high-intensity loads. However, subjective intensity should be “somewhat hard” or higher. The results also suggest that fast walking under body weight is an exercise modality in which high-intensity load, even at a high subjective intensity, is difficult to achieve.

## Introduction

Exercise tolerance is a useful prognostic indicator in healthy individuals and patients with chronic diseases [1-4]. Therefore, maintaining and improving exercise tolerance daily is necessary for healthy longevity.

High-intensity interval training (HIIT) has been recently attracting attention as a training method to increase exercise tolerance. HIIT is a method of alternating high-intensity and low-intensity exercises, often using bicycle ergometers and treadmills [5]. Although definitions of high intensity vary and are inconsistent, it is defined as 64% or more of $\grave{V}$O_2_ (oxygen uptake) max in the health support domain [6] and 60% or more of peak $\grave{V}$O_2_ in the respiratory rehabilitation domain [7]. Milanović et al. [8] conducted a meta-analysis comparing the effects of interval training and continuous training in healthy individuals. They reported that interval training was more effective in improving $\grave{V}$O_2_ max. In addition, a comparison of the effects of moderate-intensity endurance training (MICT) at 40-60% of maximum heart rate (HR), which has traditionally been used in cardiac rehabilitation, and HIIT, showed that HIIT allows for increased exercise volume and average exercise intensity in a single session; compared to MICT, HIIT is more likely to show earlier cardiopulmonary function. It is more efficient and is as good as or better than MICT in terms of exercise achievement and satisfaction; thus, HIIT is expected to be useful in cardiac rehabilitation [9,10]. These findings suggest that HIIT is a useful exercise method for improving exercise tolerance in people with and without the disease. However, many of these training methods are facility-based; require special equipment, such as bicycle ergometers or treadmills; and lack versatility for daily training at home.

HIIT using body weight, which does not require special equipment, can be performed in any location and may be more beneficial from the perspective of exercise adherence. Although there are limited reports on HIIT at body weight, Tsuji et al. [11] recently conducted a systematic review and meta-analysis on home HIIT. They reported that home HIIT was effective in improving cardiorespiratory fitness in healthy adults and patients, with no significant differences between home HIIT and medium-intensity sustained training in improving cardiorespiratory fitness. Therefore, HIIT with body weight may be useful for whole-body endurance training. To obtain the physiological benefits from training with body weight, an appropriate exercise intensity must be achieved. HR is an objective indicator of exercise intensity in endurance training, but it is difficult to use as a daily indicator because it requires evaluation equipment. Therefore, the simplest way to set the exercise intensity is through subjective intensity, which does not require special equipment. However, since subjective intensity is, as its name implies, a subjective indicator, objectively checking whether an appropriate load is being applied is necessary to obtain physiological effects. A good indicator of this is oxygen uptake. The relationship between perceived exertion and oxygen uptake is widely known. A report by Noble et al. [12] states that, in sustained exercise with a gradual increase in intensity, physiological changes such as oxygen uptake are similar to the score expressed by the concept of subjective intensity (rate of perceived exertion, RPE). A previous study [13] reported that, in relation to exercise modalities (treadmill and bicycle ergometer) and RPE, “somewhat hard” corresponds to the anaerobic metabolic threshold (AT) for both exercise modalities. These findings suggest that exercise at a subjective intensity can be an indicator of oxygen uptake; thus, it is widely used to set exercise intensity in rehabilitation. However, it is unclear whether oxygen uptake obtained at subjective intensity in HIIT with body weight, which was employed in the present study, is obtained similarly as through sustained exercise modalities, such as treadmills and bicycle ergometers, despite their differences. Verification of the level of maximum oxygen uptake of the exercise load obtained at the subjective intensity in training with body weight could be useful for improving the physical fitness of healthy individuals and prescribing exercise for individuals with diseases.

In the present study, versatile squat, front lunge, side lunge, and fast walk exercises were selected as they are considered to be easy to perform in healthy and ill patients, referring to the types of HIIT exercises performed by Ochi et al. [14]. It was also decided to define high-intensity exercise as 60% or more of peak $\grave{V}$O_2_. The aim of this study was to explore the types of exercises that utilize subjective intensity levels and body weight to test whether it is possible to achieve 60% of peak $\grave{V}$O_2_, which is a high-intensity exercise, based on the hypothesis that exercise at the subjective intensity level of "somewhat hard" in HIIT at body weight is about 60% of peak $\grave{V}$O_2_.

## Materials and methods

Participants

The study participants were 28 university students. Recruitment was carried out by publicizing the research overview to the university students in the organization. The inclusion criteria were that the participants had to be healthy men and had agreed to participate in the study. The exclusion criteria were respiratory disease, cardiac disease, and orthopedic disease of the lower limbs, but none were applicable. In this study, only male participants were included to take into consideration gender differences in motor function. Their mean age was 21.1 ± 0.6 years; mean height was 169.9 ± 4.7 cm; mean weight was 65.1 ± 9.8 kg; and mean body mass index was 22.4 ± 2.9 kg/m^2^.

**Figure 1:**
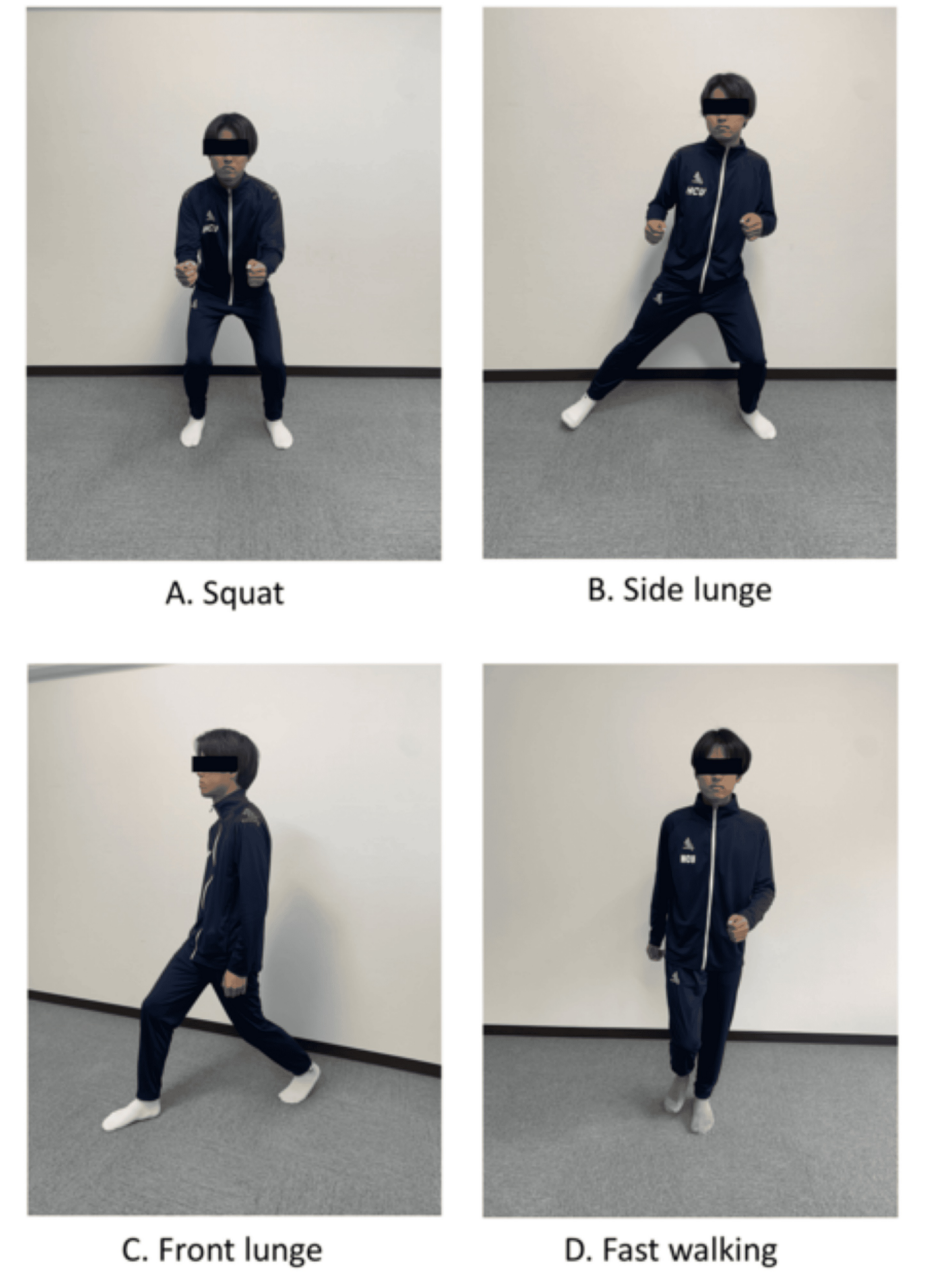
High-intensity interval training.

The sample size of 28 was calculated using the statistical power analysis software G-power (version 3.1.9.7; The G*Power Team, Germany), with an effect size of 0.25, a significance level of 0.05, and a power of 0.8. The participants were fully informed about the study both verbally and in writing and provided written informed consent prior to participation. We explained to them that participation was voluntary and that non-participation or withdrawal would not result in any disadvantages. The study was conducted in accordance with the Declaration of Helsinki and was approved by the research ethics committee of Hiroshima Cosmopolitan University (approval number 2023001).

Study design

This study used a condition-comparative cross-sectional design. Peak $\grave{V}$O_2_ was measured in a progressive exercise load test to assess the relative exercise load of HIIT. HR during high-intensity exercise, exercise frequency during HIIT, and HR during low-intensity exercise were assessed by perceived intensity.

Procedure

All HIIT exercises were performed in a standing position following the method mentioned by Ochi et al. [14], with four high-intensity exercises: squat, front lunge, side lunge, and fast walking. Squats were repeated in situ with knee flexion and extension. Upper limb flexion was encouraged for balance. Front lunge involved swinging a unilateral lower limb forward, flexing the hip and knee joints, extending and returning to the original position, and alternating between the left and right sides of the body. Side lunge consisted of swinging one lower limb out to the side, flexing the hip and knee joints, extending them, and returning to the original position; this was repeated alternately on both sides. Upper limb flexion was encouraged for balance. Fast walking was defined as walking in which the hip and knee joints were flexed and extended in situ (Figure 1). Foot stomping was considered a low-intensity exercise. In previous studies, the duration of high-intensity exercise in HIIT varied from 30 seconds to three minutes [15]. In the present study, with reference to the interval training duration mentioned by Coppoolse et al. [16], four types of high-intensity exercises were performed for one minute each, and four low-intensity foot stomps were performed for one minute 30 seconds, with low- and high-intensity exercises performed alternately for a total of 10 minutes.

HIIT exercises were performed according to the subjective intensity of the modified Borg scale (mbs) [17]. Participants were instructed to perform the exercises at a subjective intensity that felt fairly light (mbs 2), somewhat hard (mbs 4), and hard (mbs 5). The subjective intensities were measured in a random order. Low-intensity exercises were performed at a subjective intensity that felt fairly light. Before the HIIT measurements, the exercises to be performed were first explained by the examiner to the participant while performing the exercises. The depth and speed of each exercise were not specified, and the participants were encouraged to perform the exercises according to their subjective intensity. After completing the exercise at each subjective intensity, the participant rested for 10 minutes in a resting position, confirmed that the feeling of fatigue had disappeared, and then started another exercise at a different subjective intensity.

The measurements of peak $\grave{V}$O_2_ in the incremental exercise load test and each measurement during HIIT were conducted on two separate days, considering the effects of fatigue. Each measurement was performed by an examiner who had practiced and fully understood the measurement method beforehand.

Assessment

Ascending Exercise Tolerance Test

A progressive exercise load test was performed using a bicycle ergometer (Aerobike 75XL3; Konami Corp., Tokyo, Japan). The measurement was performed at a ramp load of 20 W/min, referring to the literature [18], and the number of pedal revolutions of the bicycle ergometer was maintained at 50-60 per minute. The measurements were first performed after three minutes of rest, followed by three minutes of warm-up at 25 watts; then, the load was increased and ended when the Borg index [17] at subjective exercise intensity reached 17 or the number of revolutions per minute fell below 50. Then, the peak $\grave{V}$O_2_ and maximum HR were measured. The measurements were conducted after confirming that the participants were in normal physical condition, in a quiet environment in a private room. The participants were instructed to face forward and not to speak during the measurement. The Borg index was checked by pointing to the numerical value on the Borg index form. Peak $\grave{V}$O_2_​ was measured using an expiratory gas analyzer (AE310; Minato Medical Science, Osaka, Japan). For the exhaled gas analyzer, a hot-wire flowmeter was used; gas analysis calibration and transducer calibration were conducted before each measurement to ensure that there were no problems with measurement accuracy. A maximum HR was measured using an electrocardiograph (BSM-7201; Nihon Kohden, Tokyo, Japan). For the measurement accuracy of this device, the sampling frequency is 256 Hz. A peak detection algorithm was used for heartbeat detection, and the time interval between peaks (RR interval) was calculated.

Oxygen Uptake and HR During HIIT

Oxygen uptake during exercise is an indicator of the exercise load intensity. Oxygen uptake is expressed using Fick's equation and calculated as $\grave{V}$O_2_​ = (1 pulse output x HR) x arteriovenous oxygen range. $\grave{V}$O_2_​ during HIIT was measured using an expiratory gas analyzer. HR was measured using an electrocardiograph. For high-intensity exercises, the maximum $\grave{V}$O_2_​ and HR during the four exercises were adopted, while for low-intensity exercises, the minimum of $\grave{V}$O_2_​ and HR from the second to fourth exercises were used.

Exercise Frequency

The number of repetitions was assessed using counter and video recordings during the four exercises. One squat was counted when both knees were flexed and extended, and front and side lunges and fast walking were counted as one flexion and extension movement of the unilateral lower limb. Video recordings were made with a tablet device from start to finish during four high-intensity exercise sessions. Such recordings were checked for accuracy when the accuracy of the counts on the counter raised doubts.

Statistical analyses

The statistical analysis used the maximum $\grave{V}$O_2_, HR, and exercise frequency during the four high-intensity exercises, and the average of the three lowest $\grave{V}$O_2_​​​​​​​ and lowest HR during the low-intensity exercises. First, after confirming normality with the Shapiro-Wilk test, normally distributed items were subjected to repeated measures analysis of variance; this was followed by the Tukey method as a post-hoc test. Non-normally distributed items were subjected to the Friedman test, followed by the Bonferroni method as a post-hoc test, and compared at each perceived intensity. For non-normally distributed items, effect sizes were also calculated. The exercise intensity (%$\grave{V}$O_2_) was calculated as a percentage by dividing the $\grave{V}$O_2_ by the peak $\grave{V}$O_2_ measured in the progressive exercise test.

All statistical analyses were performed using the Statistical Product and Service Solutions (SPSS, version 28; IBM SPSS Statistics for Windows, Armonk, NY), with a statistical significance level of 5%.

## Results

In the progressive exercise stress test, peak $\grave{V}$O_2_ was 2389.9 ± 611.4 mL/min, and maximum HR was 171.9 ± 16.4 beats/min.

The subjective intensity measurement results are presented in Table 1. $\grave{V}$O2 during high-intensity exercises increased significantly with increasing subjective intensity in squats, side lunges, front lunges, and fast walking (p<0.01). However, in squat and fast walking, no significant differences were found between somewhat hard and hard.

**Table 1:** Comparison of measurements by subjective intensity (n=28) *: p<0.05, **: p<0.01: fairly light vs somewhat hard; §: p<0.05, §§: p<0.01: somewhat hard vs hard; †: p<0.05, ††: p<0.01: fairly light vs hard a: After the Friedman test, the post-hoc test is performed using the Bonferroni method. Other tests are Tukey methods after repeated measures ANOVA. mean±SD, Stomping is marked as the average of the second to fourth stomping performed at fairly light intensity. METs: metabolic equivalents, CI: confidence interval

	Fairly light	Somewhat hard	Hard	Fairly light^b^ vs Somewhat hard^c^			Somewhat hard^c^ vs Hard^d^			Fairly light^b^ vs Hard^d^		
VO_2 _(mL/min)				Mean difference (c-b)	CI 95%	Effect size	Mean difference (d-c)	CI 95%	Effect size	Mean difference (d-b)	CI 95%	Effect size
(METs)												
Squat^a^	1198.8±336.8	1476.3±399.0**	1724.6±498.0††			0.71			0.33			1.04
	(5.2±1.2)	(6.5±1.8)	(7.7±2.6)									
Side lunge	1276.4±365.3	1593.6±391.7**	1853.5±441.4§§††	317.3	151.6-483.0		259.9	94.2-425.5		577.1	411.5-742.8	
	(5.6±1.3)	(7.1±1.7)	(8.3±2.3)									
Front lunge	1253.2±323.9	1587.3±331.3**	1818.1±347.8§§††	334.1	213.7-454.5		230.9	110.4-351.3		564.9	444.5-685.4	
	(5.5±1.2)	(7.0±1.5)	(8.1±1.7)									
Fast walking^a^	961.3±377.2	1200.8±459.3**	1358.0±415.5††			0.86			0.18			1.04
	(4.2±1.5)	(5.3±1.9)	(6.1±2.0)									
Stomping	586.1±190.1	669.5±179.1**	698.2±179.1††	83.4	30.4-136.4		28.7	-24.4 to 81.7		112.1	59.1-165.1	
	(2.6±0.7)	(2.9±0.8)	(3.1±0.7)									
HR (beat/min)												
Squat	120.4±15.4	128.2±16.5**	132.5±16.4††	7.8	2.3-13.2		4.3	-1.1 to 9.8		12.1	6.6-17.5	
Side lunge	125.0±16.2	137.9±19.0**	143.0±20.5††	12.9	6.4-19.4		5.1	-1.4 to 11.7		18.0	11.5-24.5	
Front lunge	126.6±16.2	140.1±19.2**	146.7±20.3§††	13.5	7.6-19.4		6.5	0.6-12.4		20.0	14.1-25.9	
Fast walking^a^	119.5±16.7	131.2±20.2**	136.4±23.1††			0.73			0.20			0.93
Stomping	102.8±17.1	109.1±19.7*	113.6±22.0††	6.4	1.2-11.5		4.5	-0.6 to 9.7		10.9	5.7-9.7	
Exercise frequency (beat/min)												
Squat	34.1±6.0	41.9±7.4**	48.3±11.8§§††	7.7	3.7-11.8		6.5	2.4-10.5		14.2	10.1-18.2	
Side lunge	27.1±4.5	32.3±6.5**	35.7±9.6§††	5.1	1.8-8.4		3.4	0.2-6.7		8.5	5.3-11.8	
Front lunge	24.7±4.9	30.1±6.0**	34.2±7.9§§††	5.4	2.4-8.4		4.1	1.1-7.1		9.5	6.5-12.5	
Fast walking^a^	146.2±33.0	165.4±31.3**	180.0±39.3††			0.66			0.39			1.05

The HR was significantly higher with increasing subjective intensity between fairly light and somewhat hard and between fairly light and hard for all high-intensity exercises (p<0.01). The values were also significantly higher for hard subjective intensity, between somewhat hard and hard, in the front lunge (p<0.05).

The number of exercise sessions increased significantly as the subjective intensity increased (p<0.01, p<0.05). However, in fast walking, no significant differences were found between somewhat hard and hard.

**Figure 2:**
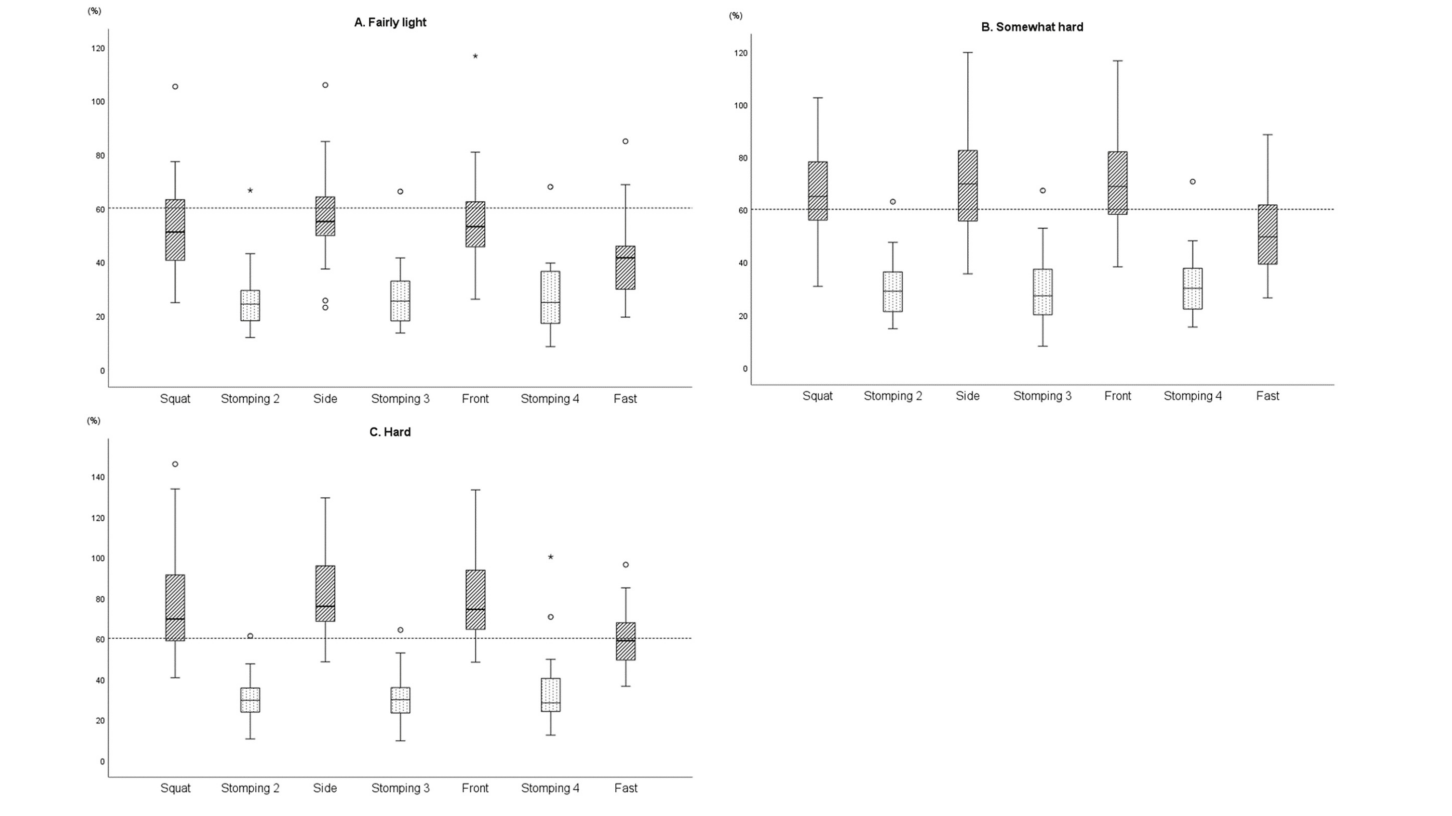
VO2 during high-intensity interval training according to subjective intensity Box-and-whisker diagrams show the analysis of the percentage of VO_2_/peak VO_2_ during high-intensity interval training (HIIT) by subjective intensity. Shaded boxes indicate high-intensity exercises, and dotted boxes indicate low-intensity exercises. For stomping, the minimum VO_2_ values were adopted when performed at fairly light intensity for the second to fourth session. Each box extends from the 25th percentile at the bottom to the 75th percentile at the top, with the median value indicated by a line across the box. The largest value is below the inner upper limit, and the smallest value is above the inner lower limit (1.5 times the box). An outer value (○) indicates that it is below or above the inner limit but within the outer limit (three times the box), while an outer value (★) indicates that it is above the outer limit (three times the box). A. Subjective intensity of fairly light. B. Subjective intensity of somewhat hard. C. Subjective intensity of hard Side, side lunge; front, front lunge; fast, fast walking

In %$\grave{V}$O_2_, more than 60% of the peak $\grave{V}$O_2_ was achieved in squats, side lunges, and front lunges at the subjective intensity of somewhat hard, but only 60% of the peak $\grave{V}$O_2_ was achieved in fast walking at hard subjective intensity (Table 2, Figure 2).

**Table 2:** Percentage of VO2 in high-interval intensity training by subjective intensity (n=28) mean±SD. For squat, side lunge, front lunge, and fast walking, the maximum VO_2_ values were adopted; for stomping, the minimum VO_2_ values were adopted when performed at fairly light intensity from the second to the fourth stomping session. %VO_2_ , Percentage of VO_2_/Peak VO_2_

	Fairly light	Somewhat hard	Hard
%VO_2_			
Squat	53.1±17.4	65.3±18.0	76.6±25.3
Side lunge	56.1±16.8	70.5±19.0	81.8±20.8
Front lunge	55.5±17.6	70.5±19.1	80.9±21.7
Fast walking	42.3±15.8	52.2±16.4	59.1±14.5
Stomping	26.3±11.3	30.0±11.8	31.7±12.3

## Discussion

The intensity of body weight exercises was assessed in healthy young male adults according to the subjective intensity. The results showed that $\grave{V}$O_2_ increased significantly with increasing subjective intensity in side and front lunges. In squat and fast walking, as with the other exercises, $\grave{V}$O_2_ tended to increase as the subjective intensity increased, but no significant differences were found between somewhat hard and hard.

As shown in Fick's formula, oxygen uptake is a comprehensive indicator of the function of the lungs, heart, and muscles and is a useful indicator of exercise intensity [19]. Single pulse output during exercise did not increase after 40% of $\grave{V}$O_2_ max as it reached a plateau [20]. Squat, side lunge, front lunge, and fast walking in the present study had %$\grave{V}$O_2_ above 40% at all subjective intensities, suggesting that the single pulse output may have reached its peak. Therefore, HR and arteriovenous oxygen calibrations are likely factors in the increase in $\grave{V}$O_2_.

With regard to HR, a significant difference was found between the somewhat hard and hard intensities only in front lunge, but not in squat, side lunge, or fast walking. These results suggest that the factors contributing to the increase in $\grave{V}$O_2_ between somewhat hard and hard, except in front lunge, may be dependent on the arteriovenous oxygen gradient. In squat and fast walking, significant differences in $\grave{V}$O_2_ were found only between fairly light and hard and between fairly light and somewhat hard, suggesting that the increase in arteriovenous oxygen gradient with increasing exercise intensity may not have been observed between somewhat hard and hard. According to Fick’s equation, the arteriovenous oxygen range is related to oxygen uptake in muscle tissue. Therefore, it may be difficult to fine-tune muscle activity by subjective intensity in squats and fast walking. Traditionally, it has been reported that the sense of fatigue and skeletal muscle activity from exercise using very high loads, such as resistance exercise, is maintained at high levels. [21,22] Additionally, Wu et al. [23] compared lower limb muscle activity between front lunge and squat with body weight and reported higher muscle activity in front lunge. Therefore, in front lunge, muscle activity and adjusting the exercise load according to subjective intensity may be easy. However, squatting and fast walking were less likely to produce higher muscle activity than front lunge, suggesting that it may be more difficult to adjust the exercise load by subjective intensity.

In terms of exercise intensity, in respiratory rehabilitation, high-intensity exercise is defined as 60% or more of the peak $\grave{V}$O_2_ [7]. In the present study, the mean values of high-intensity exercise load for each subjective intensity ranged from 42-56% for fairly light, 52-71% for somewhat hard, and 59-82% for hard. This indicated that the subjective intensity of light was not sufficient and that somewhat hard or higher was necessary to obtain a high-intensity load. In terms of load intensity according to exercise type, high-intensity loads were obtained in squats, side lunges, and front lunges, but they were difficult to obtain in fast walking at any exercise intensity. In a report on lower limb muscle activity in front lunge and squat, Wu et al. [23] reported it to be greater in front lunge than in squat. However, this was because the only load in the own-weight-bearing exercise was the body weight of the performer. Squat supports body weight with both legs, whereas front lunge requires higher lower limb muscle activity than squat, as only the body weight is loaded, and involves a floor reaction force [24], which results in a higher intensity exercise. Similarly, in a side lunge, the weight on one leg was a factor in obtaining a higher load.

In contrast, fast walking was less likely to produce a higher load than squat, front lunge, and side lunge, as reported by Iwashita et al. [25] who compared quadriceps muscle activity during squatting and walking on a treadmill. They reported a smaller increase in muscle activity in walking than in squatting. One possible difference between the two is that there was a smaller shift in the center of gravity on the treadmill. Therefore, it was assumed that, in walking movements with less center-of-gravity shift, the load due to loading on the lower limb was limited and an increase in quadriceps muscle activity was less likely. Therefore, it was considered that, in fast walking, there was a limit to the muscle activity that could be obtained even though the number of exercise sessions was increased; thus, it was difficult to identify subjective fatigue, and no significant difference in the number of exercise sessions between somewhat hard and hard was observed.

With regard to subjective intensity and exercise load, previous studies reported [13,26] that the rating of perceived exertion (RPE) at the AT level is largely unaffected by the exercise mode. In the present study, the %$\grave{V}$O2 based on RPE corresponding to “somewhat hard” ranged from 65% to 71% on average in squat, front lunge, and side lunge. Previous studies have reported a %$\grave{V}$O_2_ max corresponding to “somewhat hard” of approximately 60% [27]. In the present study, the percentage of peak $\grave{V}$O_2_ was calculated in relation to peak $\grave{V}$O_2_ in the progressive exercise stress test. Therefore, it can be inferred that the values were slightly higher compared to %$\grave{V}$O_2_ max. Therefore, the results of the present study are inferred to be in line with those of previous studies. However, in fast walking, it was difficult to obtain the load to reach the %$\grave{V}$O_2_​​​​​​​ corresponding to AT, suggesting that the relationship between subjective intensity and exercise load in fast walking differs from that in previous studies.

Limitations

In the present study, the arteriovenous oxygen gradient and lower limb muscle activity were not measured during the high-intensity exercise. Therefore, changes in these values with subjective intensity could not be verified. Additionally, the nutritional intake situation on the day was not taken into account, which may have affected oxygen intake. Since the study only included young healthy male university students, which may have introduced selection bias, we cannot rule out the possibility that the results would have differed if more diverse patients were included. Furthermore, the effects of HIIT on aerobic capacity were not tested in this study. Therefore, in the future, the training effects of HIIT should be tested longitudinally on a variety of subjects.

## Conclusions

The results of this study suggested that high-intensity loads can be obtained in squats, front lunges, and side lunges, which require a subjective intensity of “somewhat hard” or higher. The results also suggested that fast walking under body weight was an exercise modality in which it is difficult to obtain a high-intensity load, even at a high subjective intensity. By being aware of more than somewhat hard at subjective intensity, high-intensity load can be achieved in squats, front lunges, and side lunges with body weight and can be used as HIIT at home without the use of special equipment. Our results can provide a basis for future verification of the effectiveness of HIIT with own body weight. The choice of practitioner may need to consider the risk of causing joint pain due to the high load-bearing capacity of the lower limb joints.
